# Decentralized Motion Control for Omnidirectional Wheelchair Tracking Error Elimination Using PD-Fuzzy-P and GA-PID Controllers

**DOI:** 10.3390/s20123525

**Published:** 2020-06-22

**Authors:** Wafa Batayneh, Yusra AbuRmaileh

**Affiliations:** Mechanical Engineering Department, Jordan University of Science and Technology, Irbid 22110, Jordan; yaaburmaileh15@eng.just.edu.jo

**Keywords:** Omnidirectional wheel, mobile Robot, wheelchair, PD-Fuzzy, GA-Optimized PID

## Abstract

The last decade observed a significant research effort directed towards maneuverability and safety of mobile robots such as smart wheelchairs. The conventional electric wheelchair can be equipped with motorized omnidirectional wheels and several sensors serving as inputs for the controller to achieve smooth, safe, and reliable maneuverability. This work uses the decentralized algorithm to control the motion of omnidirectional wheelchairs. In the body frame of the omnidirectional wheeled wheelchair there are three separated independent components of motion including rotational motion, horizontal motion, and vertical motion, which can be controlled separately. So, each component can have its different sub-controller with a minimum tracking error. The present work aims to enhance the mobility of wheelchair users by utilizing an application to control the motion of their attained/unattained smart wheelchairs, especially in narrow places and at hard detours such as 90˚ corners and U-turns, which improves the quality of life of disabled users by facilitating their wheelchairs’ maneuverability. Two approaches of artificial intelligent-based controllers (PD-Fuzzy-P and GA-PID controllers) are designed to optimally enhance the maneuverability of the system. MATLAB software is used to simulate the system and calculate the Mean Error (ME) and Mean Square Error (MSE) for various scenarios in both approaches, the results showed that the PD-Fuzzy-P controller has a faster convergence in trajectory tracking than the GA-PID controller. Therefore, the proposed system can find its application in many areas including transporting locomotor-based disabled individuals and geriatric people as well as automated guided vehicles.

## 1. Introduction

People with disabilities exist in a large number around the world. In the United States, according to a study published by the Christopher and Dana Reeve Foundation, one person out of fifty is considered paralyzed [1]. In the United Kingdom, statistical surveys showed 40,000 paralytics increasing by one every eight hours [2]. People diagnosed with paralysis, paraplegia, hemiplegia, spinal cord injuries, and lower limb muscular disorder live with a degraded life quality due to concomitant immobility [3]. Therefore, several mobility aids have been introduced recently in the market such as: conventional and electric wheelchairs, exoskeletons, orthoses, and scooters to facilitate every-day-activity and, consequently, raise productivity. Electrical wheelchairs are preferred over the conventional ones due to the fact that the majority of users cannot apply the needed physical effort to drive the conventional wheelchairs. Milenković et al. in [4] have developed a health monitoring Android application utilizing a smart phone equipped with built-in sensors to record, capture, and process physical activities of conventional wheelchair users.

Omnidirectional robots are much preferred over the conventional ones due to the fact that such robots are robust, able to maneuver sharp curves, do pivot rotations, furthermore, there is no need to a dedicated steering mechanisms which reduce the mechanical complexities [5,6,7,8]. In the literature one can see several recent studies related to the driving mechanism design and/or the reduction in the wheel slippage and vibration for the omnidirectional robots. As an example, Yang et al. [7] proposed the design of an Omni-wheelchair with two differential driving units and achieved zero-radius turning in order to solve the problem of operating a wheelchair in narrow spaces. The design of instantaneous center of rotation (ICR)-based motion controller, for non-holonomic omnidirectional platform, addressing structural singularities and chassis’ kinematics and physical constraints was implemented by Clavien et al. [9].

On the other hand, other researchers targeted the sensing and navigation of the wheelchairs. Scanning camera with fixed markers in the surrounding environment have been used as sensing elements in localizing mobile robot for indoor applications including wheelchairs and automated-guided carts [10]. Similarly, in [11], a laser-based measurement system with other sensors has been utilized for robot collision avoidance in industrial environments.

In recent developments, human–machine interface (HMI)-based tools have been paid more attention in controlling powered wheelchairs more naturally and intuitively. For instance, myoelectric signals from the forearm as well as wrist angle and accelerations were used as inputs to a machine learning-based model to represent a user intention in moving the wheelchair [12]. The online testing of the classifier, Dendogram Support Vector Machine, showed an accuracy of 90.5%. Furthermore, brain–computer interface (BCI) has been employed in controlling six asynchronous steering commands with a success rate of 94.2% [13]. Similarly, in [14], an Electroencephalography (EEG)-based BCI approach has been utilized to navigate an electric wheelchair in four main directions. The used digital signal processing techniques resulted in 79.4% of accuracy. It is worth mentioning that the studies of [12,13,14] were tested on healthy participants while a wheelchair is usually used by disabled people. This point should be considered in future related research. Choi et al. [15] proposed a kinematic Kalman filter (KKF)-based estimation method to improve the safety and riding comfort of a joystick-interfered wheelchair.

Path control is one of the challenging research studies nowadays due to the complexity of the different systems and environment surrounding such systems. For example, in [16] the authors have developed an algorithm for precise mobile robot path tracking in off road terrain that has been updated by using the tracking error dynamics. Another study on social navigation considering a human and a robot team has been presented by the authors in [17] in which they considered social aspects when introducing various navigation strategies and compared in simulated environment by terms as the average number of robots invading the personal space and the number of robots to the person’s side, with two of them using Asymmetric Gaussian Functions (AGFs) as the person’s social zone model. Ribeiro and Conceição in [18] presented a Nonlinear Model Predictive Control depending on the image plane where simplified visual features are extracted from the path to be followed. 

Environmental surrounding is very challenging in path tracking, especially when the environment is not predicted. Dutta et al. in [19] developed a re-planning module for the Computer-Aided Lift Planning system designed at Nanyang Technological University. This system employs GPU-based parallelization approach in addition to a path planning for discrete and continuous collision detection. Another study by Tang et al. aims to facilitate information exchange for Building information modeling (BIM) assisted Building Automation System (BAS) design and operation using one of the BAS open communication protocol named Building Automation and Control Networks (BACnet) and open BIM standard Industry Foundation Class (IFC) [20].

A very challenging subject in designing robots nowadays is the lack of human–robot interactions since robots are engaged in varying environmental workplaces. Muthugala et al., in [21], developed a Fuzzy logic controller that analyzes bumping level in order to get the robot’s attention requirement to its users by displaying various colors that indicates the situation to the user in order to take the appropriate action by the users. In another study by Kouzehgar et al. [22], the authors developed a crack detection approach based on deep-learning for a modular facade-cleaning robot. A real robot was used and equipped with an on-board camera to load a live video. The authors in [23] presented an integrated system that provided detailed semantic 3D models of buildings. The system could scan and reconstruct large scenes at a high level of detail, passing through five semantic levels, to generate a detailed semantic model of the building. In another study by [24] a robot tower-crane system was developed by studying the feasibility of a laser-technology-based lifting-path tracking system to improve productivity by 9.9%–50%. A path tracking was developed by Huang et al. in [25] for two vision-guided tractors of a robotic vehicle to make the two robotic vehicles move along a guide path accurately and smoothly. 

Many wheelchair users are usually seeking independent self-support; they try to avoid asking for help of others in moving their attained/unattained wheelchairs. This research was first motivated by a junior disabled female student at Jordan University of Science and Technology (JUST). She used to store her wheelchair inside a building that was considerably far from her parking spot. She usually had to get help from her sophomore sister in order to move her unattained wheelchair from and to the parking spot almost every day, in addition to the help at home. When we asked her and some other wheelchair users about the features needed in the electric wheelchair, they showed a lot of concern regarding being self-independent, in addition to the fact that most electric wheelchairs have conventional wheels which limit maneuverability, especially when a rotation is needed. A design of a smart wheelchair was developed by a group of researchers in JUST [3]. That design was tested and evaluated by the disabled female student and it was one step toward helping such users with similar cases. The authors in [3] designed and implemented a wireless motion control system for conventional electric wheelchairs. Moreover, a Wi-Fi module was equipped to provide remote control using an Android mobile application. The system showed an easy and effective remote motion control experimentally.

In this paper, a decentralized algorithm for motion control of omnidirectional wheeled robot was used to overcome the complexity and difficulty of the conventional methods in applying to a real system is developed. The method is expressed in the body frame, where the omnidirectional mobile robot has three separated independent components of motion including rotational motion, horizontal motion, and vertical motion, which can be controlled separately with independent controllers, which makes it simple to apply and independent on accurate mathematical model of the controlled object [26]. Two methods of controlling will be tested on the three components of motion and then the optimal one of each is chosen separately to get the best overall result. Thus, this study suggests the design, implementation, and control of a uniquely reliable system that controls the motion of an electric wheelchair utilizing Omnidirectional wheels. Furthermore, the developed system will be designed to be upgradable for potential future versions. The authors believe such a system will add a huge contribution to the community.

This paper is organized as follows: Section 2 introduces the modeling and the decentralized algorithm of the Omnidirectional wheelchair. Section 3 presents the trajectory tracking controllers design including both PD-Fuzzy-P and GA-Optimized PID controller depending on the decentralized algorithm. Section 4 summarizes and discusses the simulation results. The paper is concluded in the final section.

## 2. Modeling and Decentralized Algorithm of Omnidirectional Wheelchair

In this section, a derivation for the nonlinear kinematics and dynamics equations of motion governing the Omni-wheel system in addition to the decentralized motion control are presented in the following subsections. Details of the derived equation of the omnidirectional motion are adopted from [26].

### 2.1. Omnidirectional Wheelchair Modeling

**Figure 1:**
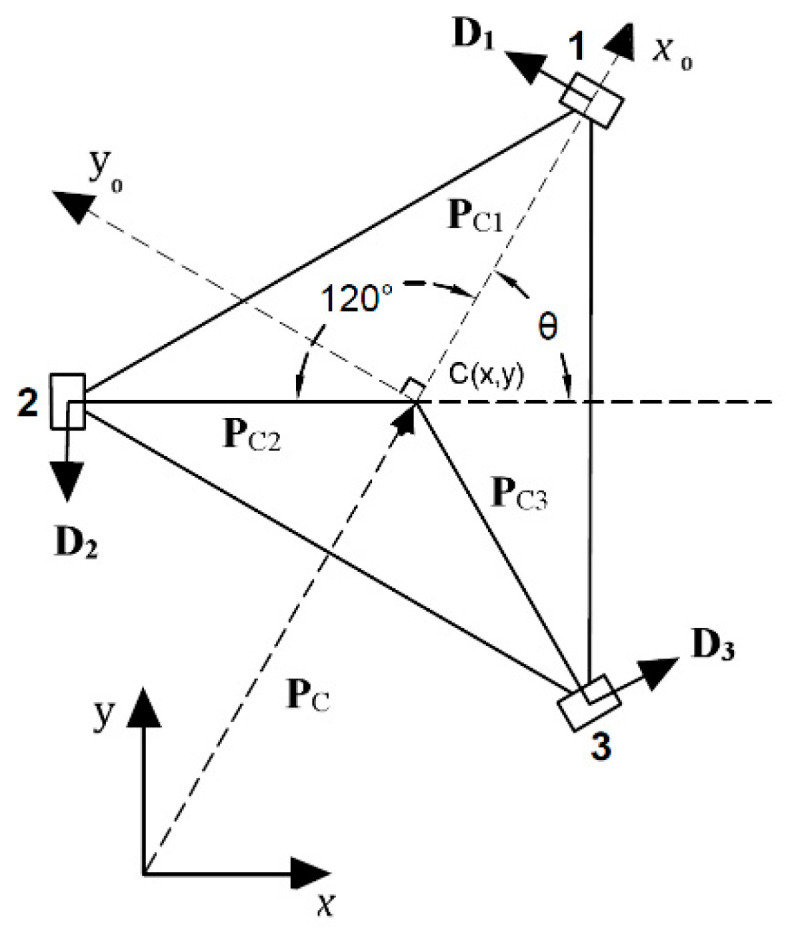
A three Omni-directional wheeled wheelchair configuration.

The proposed omnidirectional wheelchair is considered with three omnidirectional wheels spaced at 120° from one to another, it is assumed to have single rigid-body chassis with a center of geometry denoted by C. Figure 1 shows the configuration of the model, where O_xy_ represents the global coordinate frame, and C_x0y0_ represents the body coordinate frame of the wheelchair body.

The distance from each wheel’s center to the center of geometry is L, and each of the three wheels has the radius r and is driven by a separate motor. The rotation matrix is used from the body frame to the global frame as follows [26]:

(1) $$R_{1}\left(\theta \right)=\left[\begin{array}{cc} \cos\theta & -\sin\theta \\ \sin\theta & \cos\theta \end{array}\right]$$

Each wheel has a position vector **P***_Ci_* (*i* = 1,2,3) with respect to the body frame as follows:

(2) $$P_{C1}=L\left[\begin{array}{c} 1\\ 0 \end{array}\right],\;P_{C2}=R_{1}\left(2\pi /3\right)\times P_{C1}=\left(L/2\right)\left[\begin{array}{c} -1\\ {\left(3\right)}^{1/2} \end{array}\right],\;P_{C3}=R_{1}\left(4\pi /3\right)\times P_{C1}=-\left(L/2\right)\left[\begin{array}{c} 1\\ {\left(3\right)}^{1/2} \end{array}\right]$$

Likewise, the unit vectors that specify the wheels’ drive direction vectors with respect to the body frame **D***_i_* (*i* = 1,2,3) are given by:

(3) $$\begin{array}{l} D_{i}=\left(1/L\right)R_{1}\left(\pi /2\right)\times P_{Ci}D_{1}=\left[\begin{array}{c} 0\\ 1 \end{array}\right],\;D_{2}=-\left(1/2\right)\left[\begin{array}{c} {\left(3\right)}^{1/2}\\ 1 \end{array}\right],\\ D_{3}=\left(1/2\right)\left[\begin{array}{c} {\left(3\right)}^{1/2}\\ -1 \end{array}\right] \end{array}$$

The omnidirectional wheelchair’s wheels’ position vectors, **P***_i_*, and their linear velocity vectors, **v***_i_*, expressed in the global frame are obtained as follows:

(4) $$P_{i}=P_{C}+R_{1}\left(\theta \right)P_{Ci}$$

(5) $$v_{i}={\dot{P}}_{C}+{\dot{R}}_{1}\left(\theta \right)P_{Ci}$$

So, the angular velocity of wheel *i* can be presented as:

(6) $$\omega_{i}=\left(1/r\right)v_{i}^{T}R_{1}\left(\theta \right)D_{i}$$

Substituting **v***_i_* from Equation (5) into Equation (6) yields:

(7) $$\omega_{i}=\left(1/r\right)\left[{\dot{P}}_{C}^{T}R_{1}\left(\theta \right)D_{i}+P_{Ci}^{T}{\dot{R}}_{1}^{T}\left(\theta \right)R_{1}\left(\theta \right)D_{i}\right]$$

The angular velocity vector of the three omnidirectional wheelchair’s wheels is denoted by $\omega =\left[\begin{array}{ccc} \omega_{1} & \omega_{2} & \omega_{3} \end{array}\right]$, Note that a vector $q={\left[\begin{array}{ccc} x & y & \theta \end{array}\right]}^{T}$ describes the posture of the wheelchair refers to the global frame. Rearranging Equation (7) to obtain the kinematic equation expressed in the global frame as the following:

(8) $$\omega =\left(1/r\right)\left[\begin{array}{ccc} -\sin\theta & \cos\theta & L\\ -\sin\left(\pi /3-\theta \right) & -\cos\left(\pi /3-\theta \right) & L\\ \sin\left(\pi /3+\theta \right) & -\cos\left(\pi /3+\theta \right) & L \end{array}\right]\left[\begin{array}{c} \dot{x}\\ \dot{y}\\ \dot{\theta } \end{array}\right]=\left(1/r\right)A\left(\theta \right)\dot{q}$$

(9) $$\dot{q}=rA^{-1}\left(\theta \right)\omega_{C}$$

where $\dot{q}$ is the velocity vector of the omnidirectional wheelchair in the global frame.

**Figure 2:**
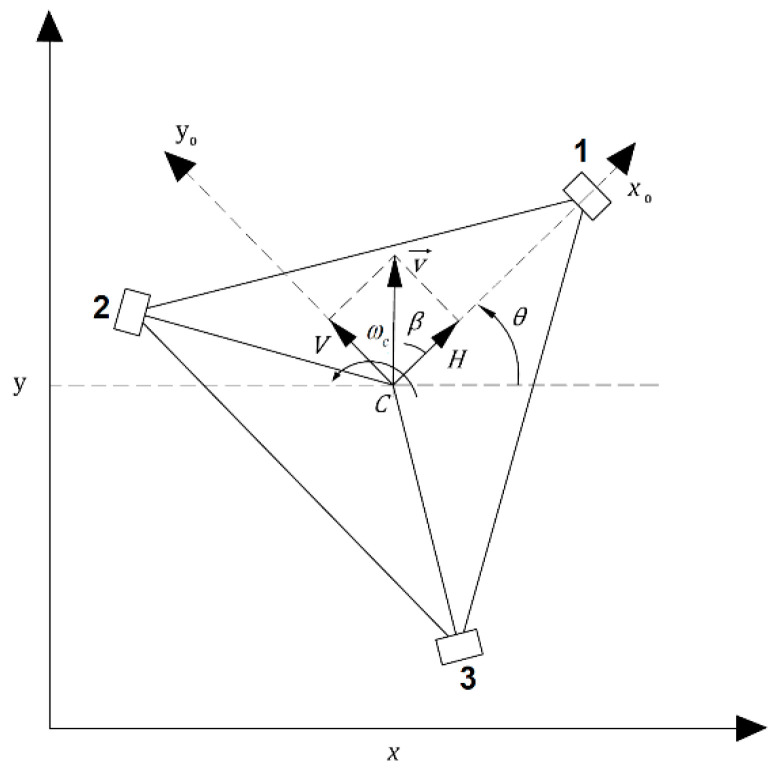
Configuration of velocity vector of the omnidirectional wheeled wheelchair in the body coordinate frame.

The motion of omnidirectional wheeled wheelchair in the decentralized algorithm should be presented in the body frame. Figure 2 shows the three separated components of the omnidirectional wheeled wheelchair motion presented in the body frame, which are rotational motion; *ω_C_*, horizontal motion; *H*, and vertical motion; *V*. These three components represent the velocity vector of the wheelchair with respect to the body frame, which is mentioned by **u** = [*H V ω_C_*]^T^.

It is easier to transform the system from global frame to body frame as the following:

(10) $$\dot{q}=R_{2}\left(\theta \right)u$$

where $R_{2}=\left[\begin{array}{ccc} \cos\theta & -\sin\theta & 0\\ \sin\theta & \cos\theta & 0\\ 0 & 0 & 1 \end{array}\right]$ is the rotation matrix.

By rearranging Equation (8) and Equation (10), yields:

(11) $$\omega =\left(1/r\right)A\left(\theta \right)R_{2}\left(\theta \right)u$$

And by simplifying **A**(θ)**R**_2_(θ) in Equation (11), yields:

(12) ω=(1/r)Bu

where $Β=A\left(\theta \right)R_{2}\left(\theta \right)=\left(\begin{array}{ccc} 0 & 1 & L\\ -{\left(3\right)}^{1/2}/2 & -1/2 & L\\ {\left(3\right)}^{1/2}/2 & -1/2 & L \end{array}\right)$ is a full rank constant matrix.

### 2.2. Decentralized Algorithm of Omnidirectional Wheelchair for Motion Control

**Figure 3:**
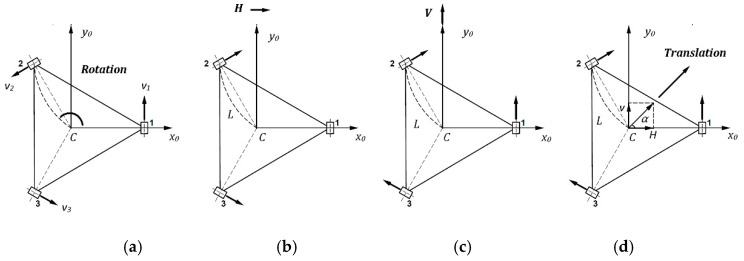
Three moving modes of omnidirectional wheeled wheelchair, (**a**) Rotational moving, (**b**) Horizontal moving, (**c**) Vertical moving, and (**d**) A translation moving is a vector sum of V and H.

The omnidirectional wheeled wheelchair has more powerful movement than a conventional wheeled wheelchair. It is considered as a holonomic robot, where at any given instant it has three controllable degrees of freedom (DOF) in its motion plane. The decentralized algorithm of omnidirectional wheelchair for motion control is used in the body frame to control the three DOF separately, which makes it simple to apply and independent on accurate mathematical model of the controlled object. As shown in Figure 3 the four moving manners of omnidirectional wheeled wheelchair: rotational motion (a), horizontal motion (b), vertical motion (c), and a translation moving (d). Details for the decentralized algorithm can be found in Reference [26].

For the first case, the pure rotational motion is attained when *ω_C_* ≠ 0 and *H = V* = 0.

(13) $$\omega_{1}=\omega_{2}=\omega_{3}=\left(L/r\right)\omega_{C}$$

Similarly, a horizontal motion is attained when *H* ≠ 0 and *ω_C_ = V* = 0

(14) $$\left\{ \begin{array}{l} \omega_{1}=0\\ \omega_{2}=-{\left(3\right)}^{1/2}H/2r\\ \omega_{3}={\left(3\right)}^{1/2}H/2r \end{array}\right.$$

and finally, a pure vertical motion is attained when *V* ≠ 0 and *ω_C_ = H* = 0

(15) $$\left\{ \begin{array}{l} \omega_{1}=V/r\\ \omega_{2}=-V/2r\\ \omega_{3}=-V/2r \end{array}\right.$$

Figure 3d shows the wheelchair moves in a combined manner (horizontal and vertical motion), it is called a pure translation motion where the motion direction is specified by the sum of V and H vectors. This motion attained when *V* ≠ 0, *H* ≠ 0 and *ω_C_ =* 0.

(16) $$\left\{ \begin{array}{l} \omega_{1}=V/r\\ \omega_{2}=-{\left(3\right)}^{1/2}H/2r-V/2r\\ \omega_{3}={\left(3\right)}^{1/2}H/2r-V/2r \end{array}\right.$$

The following equation gives the moving translation angle:

(17) α=atan(V/H)

The last case is when the three components of omnidirectional wheeled wheelchair’s motion are not zero, this mode is called simultaneous translation-rotation, and could be obtained using Equation (12). The obtained motion of the omnidirectional wheeled wheelchair using the decentralized algorithm is more flexible and effective than the conventional wheeled wheelchair.

## 3. Trajectory Tracking Controllers Design for the Omnidirectional Wheelchair

In this section, two types of controllers are designed for the omnidirectional wheeled wheelchair, namely; PD-Fuzzy-P and GA-PID controllers. Each of them has three sub-controllers for the three motion components (*ω_C_*, *H* and *V*) of the wheelchair separately, instead of using a multi inputs-multi outputs system (MIMO) controller. Then a comparison between the two approaches will be made for each component to choose the best approach for each motion. In this paper the decentralized algorithm is used to control the motion of the wheelchair under consideration where each input could be controlled independently. The tracking error in body coordinate frame can be represented as:

(18) $$e_{b}=R_{2}\left(-\theta \right)e_{g}=R_{2}\left(-\theta \right)\left(q_{ref}-q\right)$$

where $e_{b}={\left[\begin{array}{ccc} e_{H} & e_{V} & e_{\theta } \end{array}\right]}^{T}$ represents the tracking error vector in the body coordinate frame, $e_{g}={\left[\begin{array}{ccc} e_{x} & e_{y} & e_{\theta } \end{array}\right]}^{T}$ represents the tracking error vector in the global coordinate frame, $q_{ref}={\left[\begin{array}{ccc} x_{r} & y_{r} & \theta_{r} \end{array}\right]}^{T}$ is the reference pose vector and $q={\left[\begin{array}{ccc} x & y & \theta \end{array}\right]}^{T}$ is the current pose vector of the omnidirectional wheeled wheelchair.

Many industrial plants are very complicated due to different reasons, including time delays, higher order, and nonlinearities, and consequently it has been not easy to be able to tune PID controller gains accurately due to these difficulties. To overcome these complexities, modified conventional PID controllers have been produced to improve the conventional methods in tuning the PID controller parameters [27,28]. Thus, two approaches are presented in this paper to tune the PID controller’s gains; PD fuzzy to tune a proportional controller and a Genetic Algorithm (GA) to tune a PID controller. These two controllers will be developed in the following subsections.

### 3.1. PD Fuzzy-P Controller (PD Fuzzy for Proportional Adaptation)

**Figure 4:**
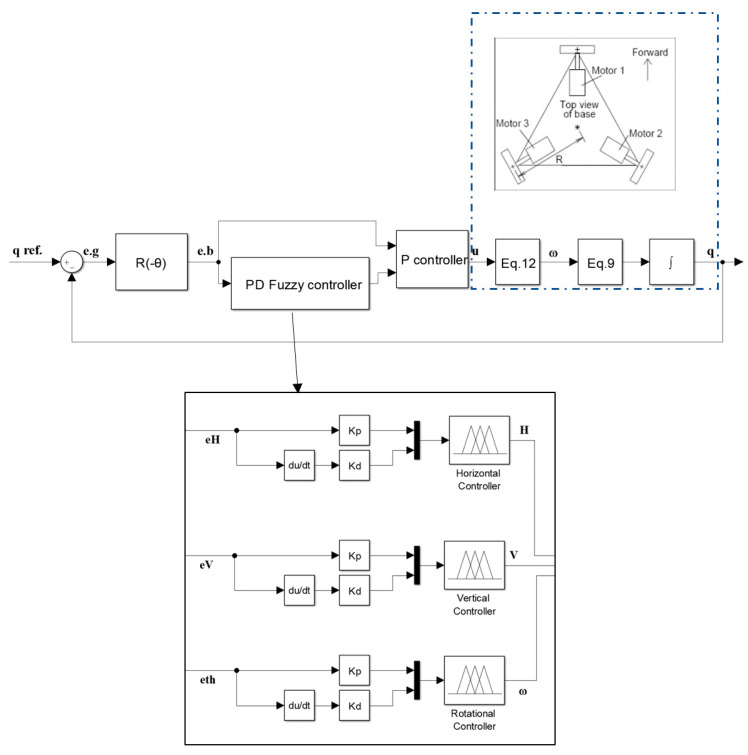
PD-Fuzzy-P controller using decentralize algorithm.

PID controller is well known and commonly used in many industries, but it does not obtain reasonable performance over a range of process conditions. Fuzzy logic is a technique in control system which deals with if-else logic, its concept was proposed by Zadeh [29], it is insensitive to parametric uncertainty, load and parameter fluctuations, and able to manage trouble with inaccurate data. Adaptation of PID using fuzzy (Fuzzy-PID) has been used in the research [30,31] because of its big advantage over the conventional PID. Thus, in this study a proportional-derivative fuzzy (PD-fuzzy) controller is used to tune a proportional controller for tracking a trajectory of omnidirectional wheeled wheelchair. For this system three separate PD-fuzzy sub-controllers are united to produce the overall controller. As shown in Figure 4, the first sub-controller is used to develop the gain of the proportional controller of the horizontal motion. Its inputs are the horizontal error e_H_ and its derivative, and its output is the change in the horizontal proportional controller gain dKp. Similarly, for the vertical and rotational motion controllers. 

**Figure 5:**
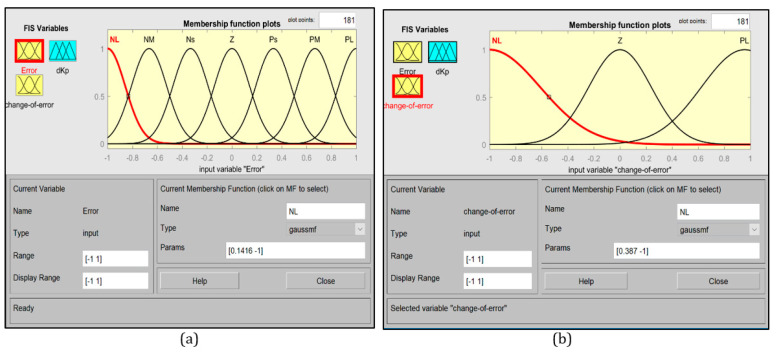
Membership functions. (**a**) e_H_, e_V_, dKp, (**b**) de_H_, de_V_.

**Figure 6:**
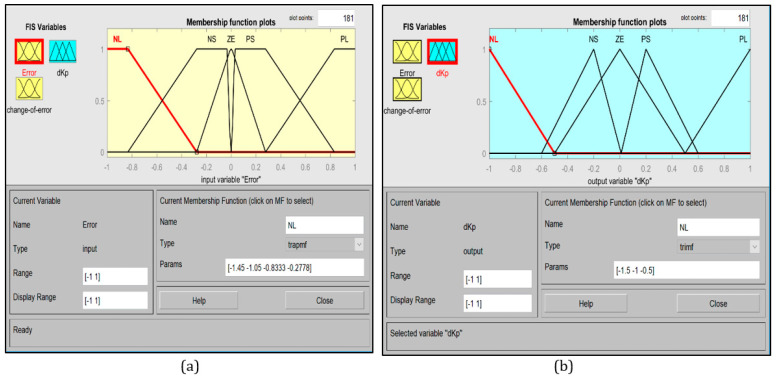
Membership functions. (**a**) e_θ_, de_θ_, (**b**) ω_C_.

The fuzzy rules are arranged to update the proportional controller gain based on the change in error and its derivative at each step. Each sub-controller will have two inputs and one output. The inputs and outputs ranges are normalized to the range of −1 to 1. Figure 5 shows the membership function plots for the horizontal and vertical motions, and Figure 6 shows the membership function plot for the rotational motion.

Table 1 shows the fuzzy law rules used for the Horizontal and Vertical controllers, and Table 2 shows the rules of the rotational controller in the form of If-then rule. For example, if **E** is NL and **DE** is NL then **dKp** is PL, where NL represents the Negative Large range, NM represents the Negative Medium range, NS represents the Negative Small range, Z represents the Zero range, PS represents the Positive Small range, PM represents the Positive Medium range, and finally PL represents the Positive Large range.

### 3.2. GA-PID Controller

The Genetic Algorithm (GA) is one of the artificial intelligence fields that belongs to the evolutionary algorithms; a stochastic global search method that mimics the process of natural evolution. It efficiently determines global minima/maxima of linear or nonlinear problems by depending on bio-inspired operators such as mutation, crossover and selection. The function to minimized is called the objective function, which contains *n* number of variables. At first an initial population of individual solutions (a vector of *n* variables) is generated and then goes through three principles to create the next generation based on the current population. These principles are listed as follows:

1. Selection: chooses the individuals (parents), these individuals produce the population of the next generation.
2. Crossover: arranges new children for the next generation by merging two old parents.
3. Mutation: forming new children by making random modulation to the new individuals.

Each individual of the new generation is applied in the fitness function, the individuals that attain the best fitness values have more chance to survive. The old generation passes away and produces a new generation with size n. In this study, GA is used to tune the PID controller gains (Kp, Ki, and Kd), which are the three variables in the objective function exist in the error of the three motion components. The objected function is formulated in the form of:

$$\mathrm{Minimize}\;M=\sum_{0}^{n}\left(q_{ref}-q\right)$$

where n is number of the wheelchair steps.

**Figure 7:**
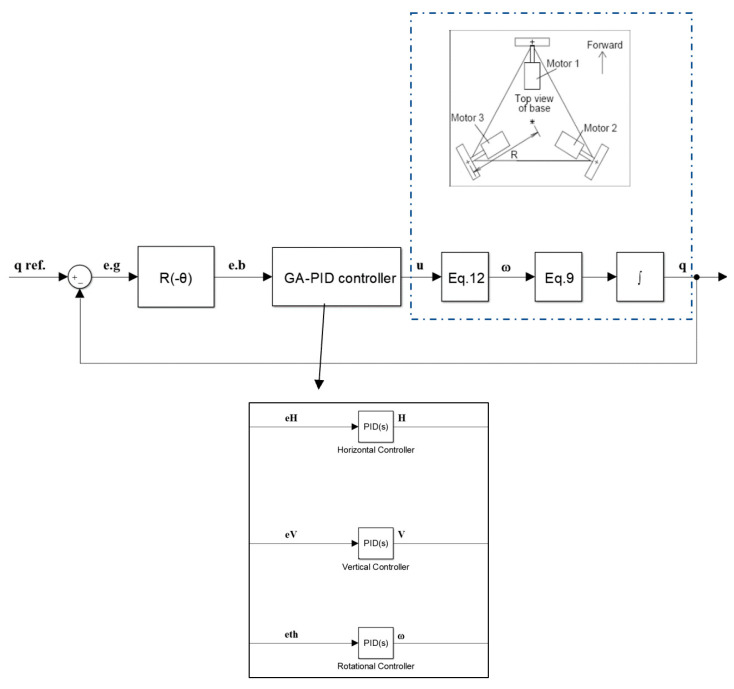
GA-PID controller based on decentralize algorithm.

The GA convergence is a user-defined specification (e.g, solution fitness threshold or the maximum number of generations). The parameters that are used in the study are specified as shown in Table 3:

Figure 7 shows conventional PID sub-controllers with its parameter optimized by GA.

It is easy to understand the GA concept and it deals with multi-objective optimization and is good for noisy environments. However, also, it has no guarantee of finding global minima.

## 4. Results and Discussion

This section presents the performance of the two approaches in different paths such as squared, circular, and rose-shaped paths. The simulation tests of design and implementation of the tuned PID based path tacking are discussed. The design of the omnidirectional wheelchair consists of three wheels spaced at 120° from one to another. MATLAB software is used for programming the system.

**Figure 8:**
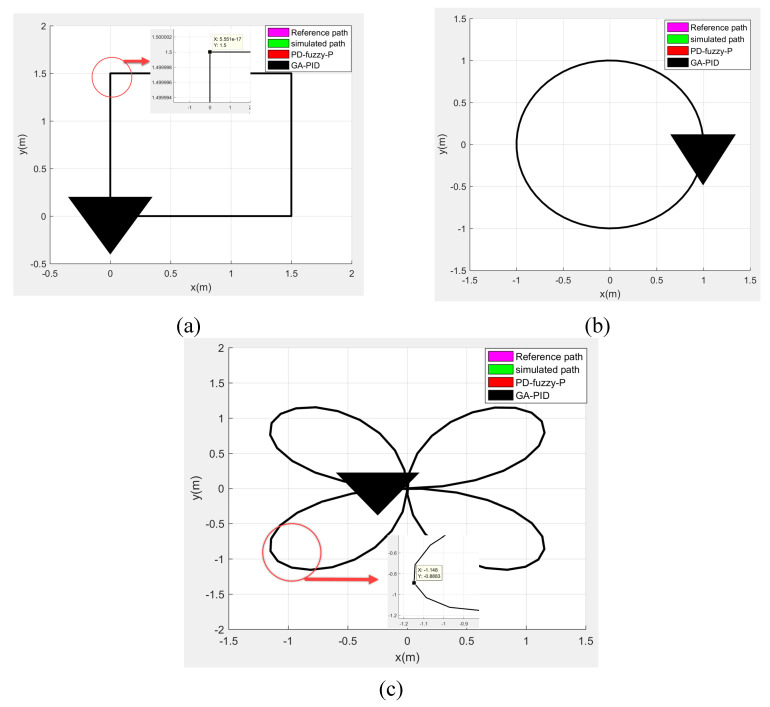
The result of the tracked trajectories: (**a**) is the squared path, (**b**) is the circular path, and (**c**) is the rose path.

The wheelchair initial conditions at starting point are x = 0 m, y = 0 m, θ =30 deg. Figure 8 shows the results of the simulated path without controller and the system response using PD-fuzzy-P and the GA-PID approaches in the squared path, circular path, and rose path in the global coordinate frame, where each side of the square is 1.5 m, the radius of the circle is 1 m, and the rose has 4 petals. The performance shows that the omnidirectional wheelchair can overcome the complexity of the sharp curves’ maneuverability, which improves the quality of life of disabled users by facilitating their wheelchairs’ maneuverability especially at the complex regions as mentioned before. In the case of square shape movement shown in Figure 8a, the zoom in view in the square shape’s corner shows how easily the omnidirectional wheelchair can track a 90˚ corner with a zero-radius turning. As well as the circular and rose shaped paths, the zoom in view in the rose shaped path’s curve in Figure 8c, shows how easily can the omnidirectional wheelchair track a U-turn with a quite small error. The PD-Fuzzy-P and GA-PID controllers’ parameters are tabulated in Table 4, where both methods attended to present a proportional controller.

The parameters of the GA used for GA-PID controller were mentioned in Table 3 and the parameters of Fuzzy controller were mentioned in Figure 6.

**Figure 9:**
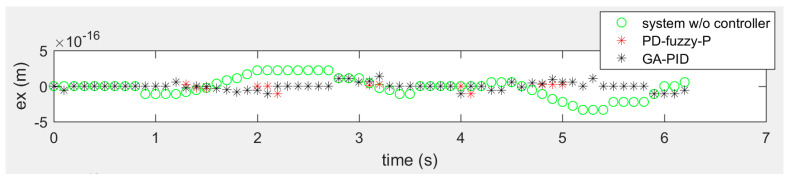
The error of the horizontal motion of the rose shaped path in the global frame in meter.

**Figure 10:**
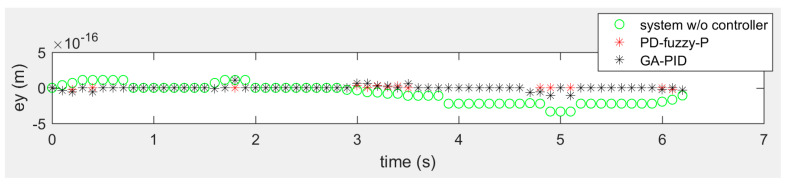
The error of the vertical motion of the rose shaped path in the global frame in meter.

**Figure 11:**
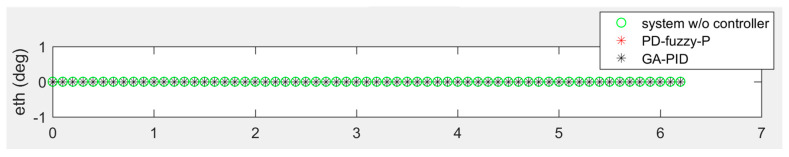
The error of the rotational motion of the rose shaped path in the global frame in meter.

Figure 9, Figure 10 and Figure 11 show the tracking errors of the omniwheeled wheelchair in the rose shaped path. Figure 9 shows the horizontal motion tracking error of the omniwheeled wheelchair without controller, with PD-Fuzzy-P and with GA-PID, where the overall error of the both approaches is about zero. PD-Fuzzy-P has the best performance with error within ±1 × 10^−16^ m around zero, and the GA-PID error is within ±2 × 10^−16^ m around zero. Figure 10 shows the vertical tracking error of the omniwheeled wheelchair without controller, with PD-Fuzzy-P and with GA-PID. Where the overall error of the both approaches is about zero. PD-Fuzzy-P has the best performance with error within ±6 × 10^−17^ m around zero, and the GA-PID error is within ±2 × 10^−16^ m around zero. Figure 11 shows the rotational tracking error of the omniwheeled wheelchair without controller, with PD-Fuzzy-P and with GA-PID, where the overall error of both approaches is zero.

**Figure 12:**
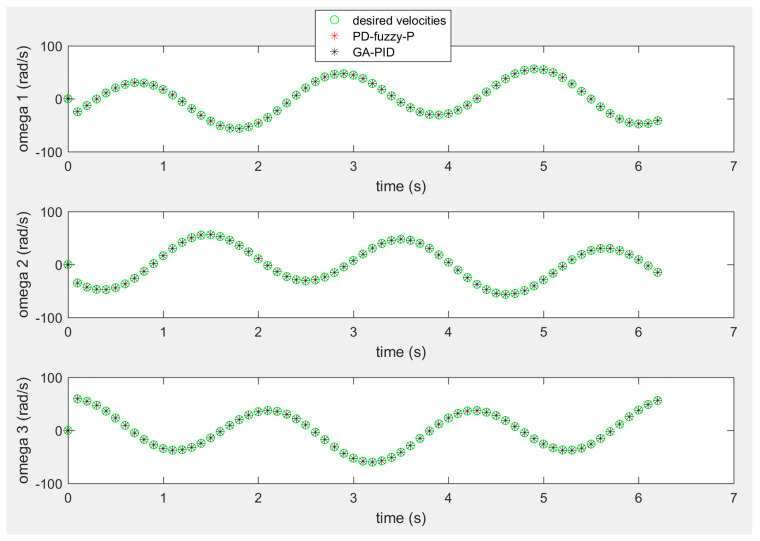
The angular velocities of the omnidirectional robot’s wheels of the rose shaped path.

The angular velocities of the three wheels in the rose shaped path are shown in Figure 12, where the obtained angular velocities from the two approaches are pretty much identical to the desired angular velocities. 

The results show that PD-Fuzzy-P controller has the best performance, for more calculations, Table 5 shows the Mean Error (ME) and the Mean Square Error (MSE) of the system are, where they are obtained as follows:

The Mean Error

(19) $$ME=\frac{1}{n}\sum_{i=1}^{n}NE_{i}$$

The Mean Square Error

(20) $$MSE=\frac{1}{n}\sum_{i=1}^{n}{\left(NE_{i}-ME\right)}^{2}$$

where the norm of the error (NE) is:

(21) $$NE_{i}={\left(e_{xi}{}^{2}+e_{yi}{}^{2}\right)}^{1/2}$$

## 5. Conclusions

This work used the decentralized algorithm to control the motion of omnidirectional wheelchairs. In the body frame of the omnidirectional wheeled wheelchair there are three separated independent components of motion including rotational motion, horizontal motion, and vertical motion, which can be controlled separately. So, each component can have its different sub-controller with a minimum tracking error. As shown in the results, the performance of this algorithm itself without controller was excellent. Two approaches of artificial intelligent-based controllers (PD-Fuzzy-P and GA-PID controllers) were designed to optimally enhance the maneuverability of the system. MATLAB software was used to simulate the system and calculate the Mean Error (ME) and Mean Square Error (MSE) for various scenarios of motion in both approaches.

The proposed system was tested in different trajectory scenarios including: squared, circular and rose trajectories. The results showed that the PD-fuzzy proportional controller has a faster convergence in trajectory tracking than the GA-PID controller in all scenarios. In addition, the omnidirectional robot showed the ability to overcome the complexity of the sharp curves’ maneuverability, such as a 90˚ corner in the square shape and a U-turn in the rose shaped path with infinitesimal error using both approaches with the superiority of the PD-Fuzzy-P controller resulted on the best performance on the system.

## Figures and Tables

**Table 1:** Fuzzy rules used for the Horizontal and Vertical controllers.

		Error						
		NL	NM	NS	Z	PS	PM	PL
Change of error	NL	PL	PM	PS	PS	NS	NM	NL
	ZE	PL	PM	PS	Z	NS	NM	NL
	PL	PL	PM	PS	NS	NS	NM	NL

**Table 2:** Fuzzy law rules used for the rotational controller.

		Error				
		NL	NS	ZE	PS	PL
Change of error	NL	PL	PL	PL	PS	ZE
	NS	PL	PL	PS	ZE	NS
	ZE	PL	PS	ZE	NS	NL
	PS	PS	ZE	NS	NL	NL
	PL	ZE	NS	NL	NL	NL

**Table 3:** Parameters of the GA used for GA-PID controller.

Characteristics	Items
population type	double vector
the population size	50
Number of variables	3 (Kp, Ki and Kd)
Selection	SUS
The cross over fraction	0.8

**Table 4:** Parameters of the PD-Fuzzy-P and GA-PID controllers.

Parameter	PD-Fuzzy-P	GA-PID
Kp	100.0457	100.3245
Ki	-	0.0050
Kd	-	0.0111

**Table 5:** The Mean Error and the Mean Square Error for both approaches in squared, circular, and rose trajectories.

	Squared		Circular		Rose	
	ME	MSE	ME	MSE	ME	MSE
PD-fuzzy-P	6.0579 × 10^−18^	5.4861 × 10^−35^	8.0953 × 10^−18^	2.7475 × 10^−34^	3.6828 × 10^−17^	2.1026 × 10^−33^
GA-PID	1.2515 × 10^−17^	2.6564 × 10^−34^	9.4170 × 10^−18^	3.1733 × 10^−34^	4.1683 × 10^−17^	2.5247 × 10^−33^
